# Plunge-diving into dynamic body acceleration and energy expenditure in the Peruvian booby

**DOI:** 10.1242/jeb.249555

**Published:** 2024-11-20

**Authors:** Francis van Oordt, Jaime Silva, Allison Patterson, Kyle H. Elliott

**Affiliations:** ^1^ Department of Natural Resources, McGill University, Montreal, QC, Canada, H9X 3V9; ^2^ Universidad Nacional Agraria La Molina, 012 La Molina, Lima, Peru

**Keywords:** Field metabolic rate, Daily activity patterns, Flight costs, Accelerometry, Doubly labelled water

## Abstract

Daily energy expenditure (DEE) is the result of decisions on how to allocate time among activities (resting, commuting and foraging) and the energy costs of those activities. Dynamic body acceleration (DBA), which measures acceleration associated with movement, can be used to estimate DEE. Previous studies of DBA–DEE correlations in birds were carried out on species foraging below their thermoneutral zone, potentially decoupling the DBA–DEE relationship. We used doubly labelled water (DLW) to validate the use of DBA on plunge-diving seabirds, Peruvian boobies (*Sula variegata*), foraging in waters above their thermoneutral zone (>19°C). Mass-specific DEE_DLW_ in boobies was 1.12 kJ day^−1^ g^−1^, and higher in males than in females. DBA alone provided the best fitting model to estimate mass-specific DEE_DLW_ compared with models partitioned per activity and time budget models. Nonetheless, the model parametrizing activity at and away from their onshore breeding colony was the most parsimonious (*r*=0.6). This *r* value, although high, is lower than that of all other avian studies, implying that temperature is not the main cause of DBA–DEE decoupling in birds. Time at the colony (∼80% of the day) was the largest contributor to DEE as it was the most time-consuming activity and involved nest defence. However, foraging was the most power-consuming activity (4.6 times higher activity-specific metabolic rate than resting at the colony), and commuting flight was higher than in other gliding seabirds. In short, DBA alone can act as a proxy for DEE, opening avenues to measure the conservation energetics of this seabird in the rapidly changing Peruvian Humboldt Current system.

## Teaser

**Summary:** Use of activity-specific energy expenditure in free-ranging Peruvian boobies, estimated using doubly labelled water, and accelerometry to validate dynamic body acceleration as a proxy measure for field metabolic rate.

## INTRODUCTION

Energy balance in wild animals determines important outcomes in their life history, such as migration patterns (Guillemette et al., 2012), breeding strategies (Dunn et al., 2018; Viera et al., 2011), growth rates (Navarro et al., 2015) and ultimately survival (Daunt et al., 2007; Elliott et al., 2014). These linkages occur because energy balance affects how an animal decides which activities to perform, such as foraging, commuting or resting (Nagy, 1987; Nagy et al., 1999). These different activities have different energetics costs and those costs will determine how an individual allocates resources (Speakman, 1997). Flapping flight is energetically expensive compared with other forms of locomotion, and this is especially consequential for seabirds that often need to travel great distances to find food at great energetic cost in very patchy environments (Ballance et al., 1997; Weimerskirch, 2007). Energy expenditure becomes especially important during high demand periods, such as breeding at a central place, or during food shortages associated with environmental change (Ashmole, 1963; Elliott et al., 2014, 2009; Tremblay et al., 2022; Welcker et al., 2010).

The estimation of energy expenditure in the wild (‘field metabolic rate’) has been a difficult task in ecological research (Elliott, 2016; Speakman, 1997). Several methods have been used for this purpose such as mass loss, heart rate, accelerometry, respirometry in semi-wild conditions, thermal imagery and doubly labelled water (DLW), although they all pose certain limitations or biases (Elliott, 2016; Green, 2011; Speakman, 1997). DLW is widely used to provide accurate estimates of field metabolic rate in animals over 24–48 h (Butler et al., 2004; Shaffer, 2011; Speakman, 1997). DLW enables calculation of activity-specific field metabolic rate using a time budget approach, which can then be applied over shorter or longer time scales (Wilson and Culik, 1993). The development of small accelerometers has facilitated more accurate estimates of activity budgets improving the time budget approach (Elliott et al., 2013; Hicks et al., 2017).

Dynamic body acceleration (DBA) is the most commonly used metric in accelerometry to estimate energy expenditure directly as opposed to via time budgets (Wilson et al., 2006). DBA is calculated using tri-axial acceleration (heave, surge and sway) allowing for the measurement of fine-scale activity-specific energy costs (Chivers et al., 2015; Kokubun et al., 2015; Stothart et al., 2016). DBA is the area under the accelerometer–time curve after removing the static component associated with posture (Wilson et al., 2006). Specifically, work is equal to the integral of force (*F*) over distance (*x*) as given by ∫*F*d*x*=*mv*∫*a*d*t*=*mv*DBA, where *m* is mass, and therefore mass-specific energy expenditure at a constant speed (*v*) is proportional to DBA, provided all work is in the direction of travel (Gómez Laich et al., 2009; Stothart et al., 2016). Because locomotion makes up a substantial proportion of an animal's energy budget, DBA and the rate of oxygen consumption (*V̇*_O_2__) are often highly correlated, but the strength of the correlation (*r*) can vary from 0.6 to 0.99 (Elliott et al., 2013; Gómez Laich et al., 2009; Stothart et al., 2016). All validations of the relationship between daily energy expenditure (DEE) calculated from DLW and DBA (DEE_DLW_–DBA) in birds to date have occurred below their thermoneutral zone, often in polar or subpolar regions, and the variation in *r* may be due to thermoregulation decoupling the DEE_DLW_–DBA relationship (Elliott et al., 2013; Stothart et al., 2016; Hicks et al., 2017; Sutton et al., 2021). That is, individuals foraging in colder water may have additional energetic costs not accounted for via movement and DBA, potentially confounding the relationship. There is therefore a need to examine the DEE_DLW_–DBA relationship in tropical seabirds, foraging in environments within their thermoneutral zone.

Energy balance can be an important metric for understanding the impact of extraction pressure on wildlife (Masello et al., 2021; Yin et al., 2024). The Peruvian Humboldt Current system (PHCS) is one the most productive marine upwelling systems on the planet, once supporting 10 million tons of seabird guano prior to the collapse of the anchovy fishery and subsequent major drop in the seabird population of the system in the 1950s (Barbraud et al., 2018; Chavez et al., 2008; Jahncke et al., 2004). There is a need to understand energy limitations for these guano birds to fully understand the factors hampering their recovery (Chavez et al., 2008; Jahncke et al., 2004; Weimerskirch et al., 2012; Zavalaga et al., 2010). Among the guano birds, Peruvian boobies (*Sula variegata*), are interesting energetically because they forage exclusively during the day and are plunge-divers with high aspect ratios that do not make deep dives or significantly use their wings to propel themselves underwater (Brewer and Hertel, 2007; Gaston, 2004; Nelson, 1978; Ropert-Coudert et al., 2009, 2004; Van Oordt et al., 2018). Peruvian boobies exclusively plunge-dive, potentially reducing both flying and diving energy expenditure at the cost of reduced mobility underwater (Nelson, 1978), contrasting with gannets, which plunge-dive and also flap their wings under the water (Ropert-Coudert et al., 2009; Sutton et al., 2023). Nonetheless, DEE in gannets is higher than in many other seabird despite low flight costs (Green et al., 2009), possibly because plunging is an energetically demanding activity, especially when flapping underwater, or because they nest in dense aggregations with many aggressive interactions (Adams et al., 1991; Côté, 2000; Ropert-Coudert et al., 2009; Shaffer, 2011; Siegel-Causey and Hunt, 1981). There is a need to understand the relative costs of flying and plunge-diving in boobies to better understand the relative role of anchovy density, distance to anchovy schools or depth of anchovies in limiting the net energy gain of boobies and thus limiting their reproductive success and population growth.

To examine the DEE_DLW_–DBA relationship in a warm water, tropical species, we developed biologger validations for plunge-diving Peruvian boobies for both time budget and DBA approaches using accelerometers. Specifically, we measured energy expenditure in Peruvian boobies using DLW and accelerometry. We used the accelerometers, time–depth recorders (TDR) and GPS to classify activities into flying, foraging, at the colony and resting (away from the colony). We predicted that: (1) total daily DBA would highly correlate with total DLW-estimated mass-specific DEE given the lack of a confounding effect of temperature, and (2) DBA would better predict DLW-estimated mass-specific DEE compared with time budget approaches because DBA incorporates variation in work within activities with variable activity rates, such as plunge-diving. To understand how DEE is influenced by activity budgets, we also report time in each activity and activity-specific metabolic rates.

## MATERIALS AND METHODS

### Study site and species

We performed our study between 11 and 17 November 2019 at Guañape Norte Island (08°32′41″S, 078°57′49″W) within the Reserva Nacional Sistema de Islas Islotes y Puntas Guaneras, Peru, when sea surface temperature was roughly 20°C. Thirty-one chick-rearing Peruvian boobies (hereafter boobies), *Sula variegata* (Tschudi 1843), were captured on their nests by approaching them during the early hours of the day and using a noose pole (>3 m long). We equipped 21 of these birds with biologgers and handling time was less than 5 min, both at deployment and at recapture. Sex was determined in the field based on size and vocalizations (Zavalaga et al., 2009). All boobies were wrapped tightly in a fabric bag and measured on an electronic scale (±1 g). We attached Technosmart (Rome, Italy) axy-trek accelerometers (18 g; sampling rate: 1 GPS fix per minute, 1 Hz for pressure; 50 Hz for triaxial acceleration) to the four central tail rectrices using two zip-ties, super glue and Tesa tape. Devices, including attachment materials, weighed 30 g, ranging from 1.6% to 2.9% of the body mass of our birds, which we assumed produced minimal effects on the birds (Bodey et al., 2018; Geen et al., 2019). No abandonment was detected as a result of biologging. We used tail-mounted devices to reduce the loss of devices due to the bird's plunging behaviour, as devices detach more easily from the back (Vandenabeele et al., 2014).

This study was approved by the McGill University Animal Care Committee.

### DLW and energy expenditure calculations

Using the same birds for equilibrium and final blood samples can alter measurements of energy expenditure because of altered behaviours due to handling stress (Schultner et al., 2010). We used the single-sample method to minimize stress of the deployed birds (handling time <5 min for non-equilibrium birds), as described by Stothart et al. (2016) and well documented by Speakman (1997). Briefly, we divided our sample of 31 birds in two sets: one set for estimation of equilibrium of isotopic concentrations, and a second set on which we deployed accelerometers (as described above) and on which we calculated activity budgets.

The first set of birds consisted of 10 birds used to determine the relationship between isotopic dilution and body mass known as the equilibrium rate. We extracted blood in three capillary tubes (∼150 µl) from the brachial vein of all birds as soon as they were captured for isotopic background value readings. We injected 1 ml of DLW (50% H_2_^18^O and 50% D_2_O; see Elliott et al., 2013, for details) in the pectoralis muscle and kept the birds in a dark box for 1 h before collecting three new capillary tubes (∼150 µl) of blood from the other wing's brachial vein. All capillary tubes obtained in the field were flame sealed for later analysis. We obtained a dilution relationship equation per isotope (deuterium and oxygen-18) with high correlation values (Pearson's *r*>0.7; Fig. S1), so we were confident that using the single-sample technique for the second set of birds would provide accurate estimates of energy expenditure.

The second set of birds consisted of 21 individuals that were captured and injected with 1 ml of DLW (same mixture as described above) and released with GPS-accelerometers within 5 min of injection. All birds were recaptured ∼48 h after deployment (range 46.1–61.3 h, s.d.=3.7), except for one bird, which was recaptured ∼100 h after and was not included in the energy expenditure validation analysis because the biologger turned off before recapture. Upon recapture, we obtained a blood sample from the brachial vein in two to three capillary tubes (∼150 µl). Biologging devices were carefully removed to avoid the loss of feathers.

All blood samples were distilled in the lab through evaporation after 24 h on a hotplate while in flame-sealed glass Pasteur pipettes. DLW analysis was performed using a Los Gatos Liquid Water Isotopic EP Benchtop Analyzer (model GLA430 LWIA-912, Los Gatos Research, San Jose, CA, USA; hereafter LWIA). Samples were run later in batches of corresponding enrichment using DLW standards for each batch (high enrichment for initial samples and very low enrichment for background value samples, both from the first set of birds, and low enrichment for final samples from the second set of birds). Each run consisted of five preparation injections and five measured injections, which were averaged to obtain the final value for the H^2^/H^1^ and O^18^/O^16^ ratios for all posterior calculations. We used a low and a high standard at the beginning and end of the run, as well as one of each standard between every 2–4 samples to correct for any residual effect during the run. In the case of some unusual readings, such as low density (due to bubbles in the vials) or pressure errors, samples were rerun or redistilled when needed.

All calculations followed Speakman (1997) as described in the Appendix.

### Behavioural classification and accelerometry

**Fig. 1.**
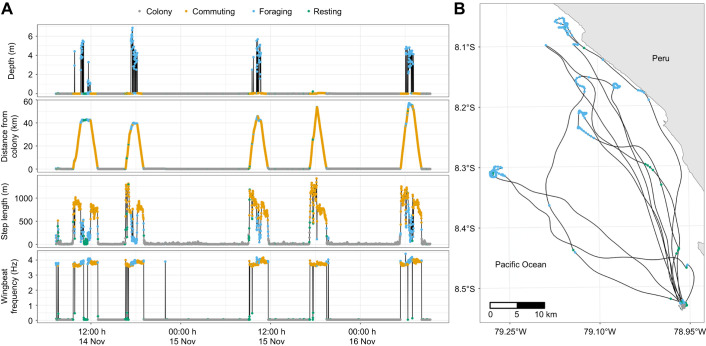
**Behavioural classification of Peruvian boobies.** (A) Sample full track of one bird with the Hidden Markov Model (HMM) behavioural classifications showing ‘colony’ (grey), ‘commuting’ (yellow), ‘foraging’ (blue) and ‘resting’ (green) for the respective values (from top to bottom) of diving depth, distance to the colony, step length and wingbeat frequency. (B) Mapped track with HMM behavioural classifications, where black lines represent ‘commuting’. HMM parameters for each behavioural class are shown in Table 1.

We used Hidden Markov Models (HMMs) with the momentuHMM package (McClintock and Michelot, 2018; Patterson et al., 2019) to classify four behavioural states of boobies: colony, commuting (exclusively flying), foraging (plunging, underwater time, lift-off; may include short-distance flying before and after the plunge), and resting (away from the colony) (see Fig. 1 for example behavioural data and a mapped track). Because this method requires a consistent sampling frequency and step length (distance travelled between GPS locations), we interpolated all tracks to 1 min sampling rates using the crawlWrap function from momentuHMM (to account for potential gaps in GPS locations). We estimated behavioural states using four data streams (presence at the colony, wingbeat frequency, diving, step length) according to the parameters show in Table 1. Briefly, we calculated metrics for all data streams at the original accelerometer rate (50 Hz), interpolating non-accelerometer data when needed (GPS locations and depth). Then we obtained a mean value by extracting the 50th quantile per minute, to thin the data for the HMM models. ‘Colony’ state was based on the distance from the colony (distances ≤0.5 km from the colony=1, distances >1 km from the colony=0); wingbeat frequency, calculated as the peak frequency of the *z*-axis over a 30 s window using a Fast Fourier Transform; ‘diving’ as the proportion of time underwater during a 1 min window according to the depth sensor (e.g. not diving during 1 min window=0, or diving for 30 s=0.5). Probability distributions for each data stream and starting values for each behavioural state used in the HMM are provided in Table 1.

**Table 1.**
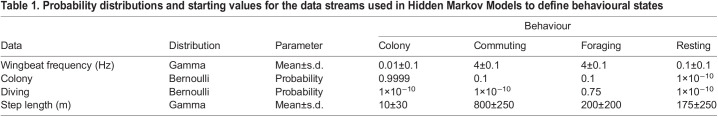
Probability distributions and starting values for the data streams used in Hidden Markov Models to define behavioural states

Vectorial dynamic body acceleration (hereafter DBA) for DEE models was calculated using:
(1)

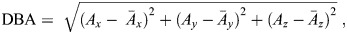
where *A_x_* is the acceleration measured every 50 Hz in the antero-posterior axis (surge), *A_y_* is the acceleration in the lateral axis (sway), *A_z_* is the acceleration in the dorso-ventral axis (heave) and *Ᾱ_n_* represents the static component of the acceleration in each axis, calculated over a 3 s running window (Shepard et al., 2008; Stothart et al., 2016). We then calculated mean DBA values over 1 s (for smoothing) and summed it over each minute of sampling to match the HMM classifications. DBA was summed and then standardized for a 24 h period for total daily DBA as well as for daily activity-specific DBA. Specifically, total daily DBA=(total DBA of deployment/total sampling time for each bird in hours)×24 h; and daily activity-specific DBA=(total DBA_activity_ of deployment/total sampling time for each bird in hours)×24 h.

### Statistical analyses

All statistical analyses were performed using R (http://www.R-project.org/). We first tested differences between sexes in total mass-specific DEE_DLW_ using linear models, as boobies are sexually dimorphic in size (body mass). No deviations from normality in the model were significant. Although there were significant differences between sexes in mass-specific DEE in boobies, we grouped all individuals after doing this comparison because of small sample sizes per sex, considering that males and females represented a continuum of body mass along the *x*-axis. Sex-specific models are presented in Table S1.

We built 10 linear regression models for all birds parametrizing activity-specific energy expenditure for commuting, foraging, resting and colony, as we expected these activities to have different energy requirements. Time budget models were based on the full model considering all activities as follows:
(2)

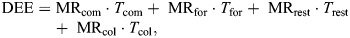
where DEE is the daily DLW-estimated energy expenditure (derived from the total sampling period); MR_com_ is commuting metabolic rate, MR_for_ is foraging metabolic rate, MR_rest_ is resting metabolic rate and MR_col_ is metabolic rate at the colony; *T*_com_ is time spent commuting, *T*_for_ is time spent foraging, *T*_rest_ is time spent resting and *T*_col_ is time spent at the colony. The intercept was set to zero when accounting for all activities performed by the individual during the allotted time, as suggested by Wilson and Culik (1993). All activity times were calculated using the classification from HMMs as described above.

DBA models were built likewise, following the full activity formula:
(3)

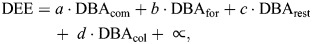
where DBA*_n_* is total DBA in each activity [commuting, foraging, resting (away from the colony) and at colony] and ∝; is the intercept (i.e. DEE with no activity, representative of basal metabolic rate, BMR). For both modelling approaches we considered combined activities in addition to independent parametrization (e.g. time commuting and foraging, as one parameter) under the assumption that the DEE_DLW_–DBA conversion parameter may be similar across some activities.

We then compared all 10 time budget and DBA models and two null models (an intercept model and a total DBA model) using the corrected Akaike's information criterion (AICc) approach (see Table 3). The AICc ranks models, penalizing them if they have an increased number of parameters without an improvement in fit, and it applies a sample size correction which additionally increases penalization. The best models were then plotted with marginal effects using the ggpredict() function from the ggeffects package (https://CRAN.R-project.org/package=ggeffects) with 95% confidence intervals.

To additionally assess the predictive capacity (Stothart et al., 2016; Sutton et al., 2023) of the best ranked models by the AICc, we investigated the correlation strength between predicted values and the actual DEE values using linear regression and reporting the correlation coefficients (*r*). We also present the mass-specific DEE (hereafter _MS_DEE_DLW_) estimation values for each activity for both approaches (time budgets and DBA) to demonstrate activity-specific metabolic rates and plot flying costs using DBA activity-specific estimates in comparison to other flying–gliding birds. As our DBA metrics were normalized to a daily basis, we avoided Halsey's time trap (Halsey, 2017). We compared time budgets between sexes using linear models (ANOVA), because boobies are highly dimorphic and our DEE estimates are mass specific, and DBA relationships with time with linear regressions (to further determine whether relationships were driven by time or DBA; Halsey, 2017). For comparative purposes we also calculated BMR using the updated allometric formula presented in McKechnie and Wolf (2004) and also compared our results with those obtained using the Seabird FMR Calculator (Dunn et al., 2018).

## RESULTS

_MS_DEE_DLW_ in Peruvian boobies averaged 1.12±0.29 kJ day^−1^ g^−1^ (mean±s.d.), and was higher in males (*t*_1,18_=4.21, *P*<0.001) (Table 2).

**Table 2.**
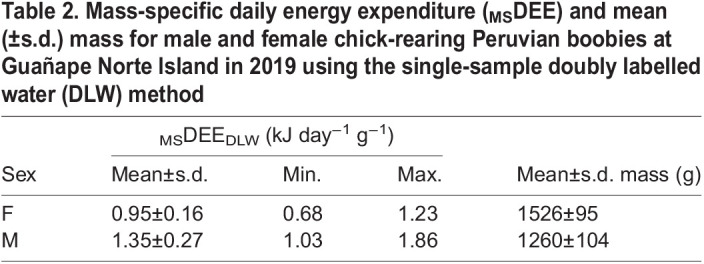
Mass-specific daily energy expenditure (_MS_DEE) and mean (±s.d.) mass for male and female chick-rearing Peruvian boobies at Guañape Norte Island in 2019 using the single-sample doubly labelled water (DLW) method

**Fig. 2.**
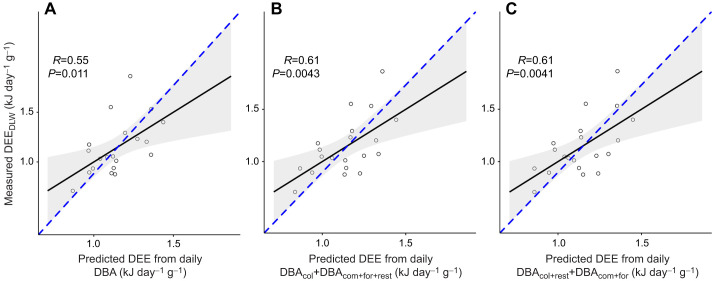
**Relationship between measured and predicted daily energy expenditure for the top three best models from the AICc ranking with the full dataset.** Daily energy expenditure from the doubly labelled water method (DEE_DLW_) and (A) predicted DEE from the model for daily dynamic body acceleration (DBA), (B) predicted DEE from the model parametrizing mean daily DBA of activity in the colony (DBA_col_) and mean daily DBA activity away from the colony (commuting, foraging and resting: DBA_com,for,rest_) and (C) predicted DEE from the model parametrizing mean daily DBA at the colony and resting and mean daily DBA commuting and foraging (*n*=20). Mass-specific DEE_DLW_ data for males and females were combined. Dashed blue line represents equal distribution of values for both axes.

Three models predicted _MS_DEE_DLW_ with ΔAICc <2. The best model to predict _MS_DEE_DLW_ was daily DBA (Table 3, *r*=0.55; Fig. S2, Table S2). The other two models had only two predictor variables: (1) DBA at colony with resting away from the colony and DBA commuting with foraging, and (2) DBA at colony and DBA away from colony (commuting, foraging and resting) (Table 3; Fig. S2, Table S2). Both two-parameter models had strong predictive capacity (*r*=0.61, *P*≈0.004) (Fig. 2). Also, activity-specific daily DBA while resting was lower compared with that of all other activities (Table S3). Total DBA at the colony was weakly correlated with time spent at the colony (*r*=0.46), which is the opposite to what we would expect due to the ‘Halsey trap’. In contrast, time spent away from the colony and DBA away from the colony were strongly correlated (*r*=0.83), suggesting that DEE in the colony is independent of time spent, and associated with specific behaviours (e.g. territoriality, thermoregulation, etc.).

**Table 3.**
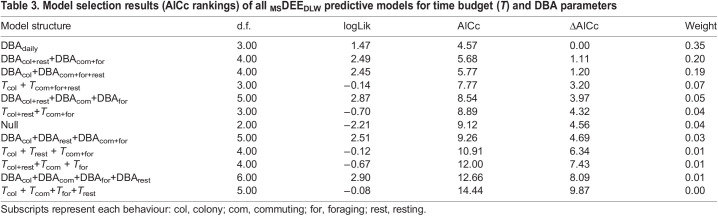
Model selection results (AICc rankings) of all _MS_DEE_DLW_ predictive models for time budget (*T*) and DBA parameters

**Fig. 3.**
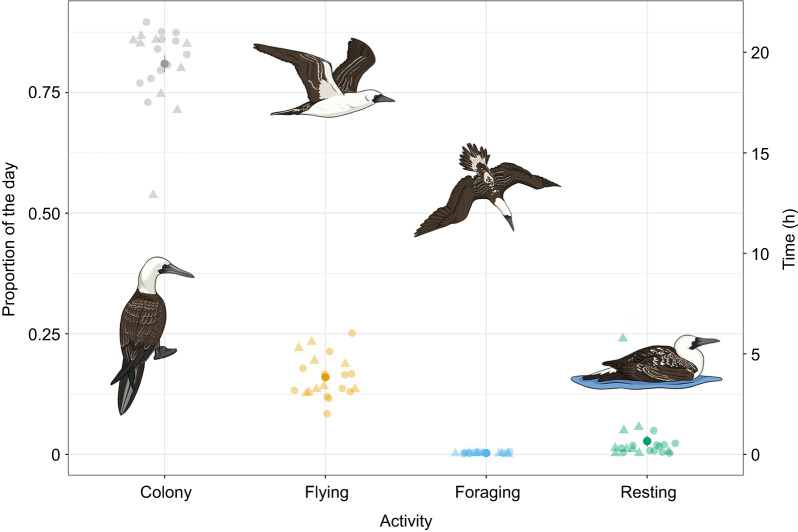
**Mean daily proportion of time spent in each activity by Peruvian boobies in Guañape Norte Island in 2019.** Filled circles represent means and semi-transparent circles (females) and triangle (males) are the individual values. Error bars represent s.e.m. (*n*=20).

Peruvian boobies averaged more than 19 h of the day in the colony and ∼3.8 h in commuting flight, with a maximum of 6 h spent commuting among the sampled birds (Fig. 3; Table S3). In both estimation approaches (time budgets and DBA), total energy spent at the colony was highest, followed by commuting and finally resting – but only because much more total time was spent at the colony (Table S3). On an hourly basis, energy costs were 2.7 times higher in commuting flight than at the colony, and 4.6 time higher when foraging (Table 4). Conversely, costs of resting away from the colony were 2.6 times higher than at the colony. Additionally, hourly DBA at the colony during the day was significantly higher than at night (*F*_1,40_=45.88, *P*<0.0001).

**Table:**
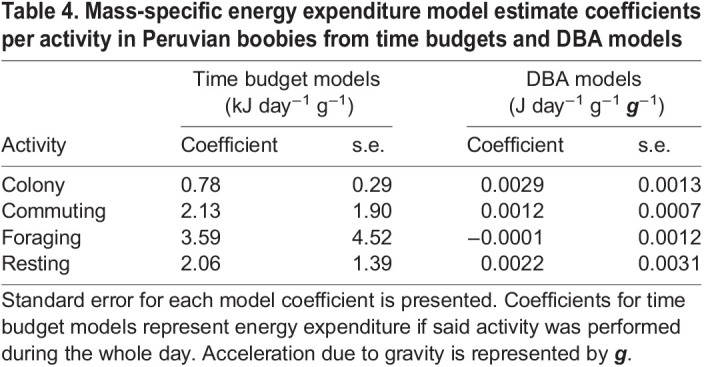
Table 4. Mass-specific energy expenditure model estimate coefficients per activity in Peruvian boobies from time budgets and DBA models

## DISCUSSION

DBA predicted DEE in a plunge-diving, flap-gliding seabird, the Peruvian booby. Interestingly, whereas most other studies have found that DBA in flight and diving partitioned separately from other activities (Ste-Marie et al., 2022; Stothart et al., 2016), in our study, the most parsimonious model predicted DEE from DBA alone, or perhaps with colony partitioned separately (Table 3). This means that DEE can be estimated in wild plunge-diving boobies from a single metric, DBA, across many different locomotory modes. Whereas Sutton et al. (2023) found that the predictive capacity in gannets increased with the incorporation of other movement metrics (e.g. total distance travelled), our DBA-only models achieved a predictive capacity within the range of their best models (from 0.6 to 0.8). Whereas all previous studies occurred in waters below the thermoneutral zone of birds, either in polar regions or in southern Australia (Elliott et al., 2013; Stothart et al., 2016; Ste-Marie et al., 2022; Hicks et al., 2020; Sutton et al., 2021, 2023), with a DEE_DLW_–DBA *r*>0.6, our study occurred in waters with sea surface temperatures of 20°C. Thus, the unexplained variance in previous studies does not appear to be directly related to thermoregulation in cold water (which would increase DEE for a given DBA). Perhaps overheating in flight or at the colony, leading to higher DEE_DLW_ but not higher DBA, is a greater source of error (Guillemette et al., 2016).

We present the first estimate of energy expenditure in Peruvian boobies using DLW. The need to calculate DEE of seabirds in the highly productive and over-exploited PHCS has been noted since the late 1960s. Our calculated energy expenditure values are similar to those of other sulids, such as red-footed boobies (∼1 kJ day^−1^ g^−1^, from Balance, 1995) and northern gannets (∼1–1.5 kJ day^−1^ g^−1^, from Birt-Friesen et al., 1989; Pelletier et al., 2020), but are slightly higher than those presented by Laugksch and Duffy (1984), who estimated energy requirements in a larger ecosystem-level analysis. Conversely, Boyd et al. (2014) modelled energy expenditure of Peruvian boobies by means of allometric equations, and found a DEE of ∼0.8 kJ day^−1^ g^−1^, much lower than our DLW direct estimation (even lower than for males alone, see Table 1). Similarly, the Seabird FMR Calculator developed by Dunn et al. (2018) estimated the Peruvian booby's field metabolic rate (FMR) for a colony at the same latitude as our study site as ∼0.9 kJ day^−1^ g^−1^, still lower than our DEE_DLW_ direct measurements, demonstrating the need for *in situ* estimates of FMRs.

During chick rearing, Peruvian boobies spent about ∼80% of their time in the colony, mainly attending their nest. Boobies spent most of their time away from the colony commuting, and individual foraging bouts were short because of their plunge diving habits (Fig. 1; see Appendix). As expected in diurnal, chick-rearing birds, time budgets showed a high bias toward time in the colony (Collins et al., 2016). Unlike in other sulids (e.g. northern gannets), foraging trips in boobies are fairly short (Carboneras, 1992; Nelson, 1978), hence the large amount of time spent at the colony, but energy expenditure values are fairly close to those of other sulid species that are present at the colony only 50% of the day (Sutton et al., 2023), implying that activity costs at the colony were higher at our study site.

The correlation between DBA and DEE despite the high time spent at the colony implies that DBA predicts DEE even when ‘resting’ at the colony. Densely populated seabird colonies like those of Peruvian boobies are prone to having intense aggressive interactions that imply higher than expected energy costs (Viera et al., 2011). Peruvian boobies, especially males, show highly aggressive behaviour toward other individuals in transit to their nests or scouting for females. This behaviour may occur in Peruvian boobies, as in other sulids, such as Nazca boobies, that commonly perform aggressive non-parental adult visits to chicks (Müller et al., 2011). This could explain why DBA models, rather than time budget models, and more especially the model with DBA in the colony as a separate parameter from other activities, ranked better at predicting _MS_DEE_DLW_. Using heart rate loggers coupled with accelerometers to further explore the periods of time at the colony when DBA and metabolic rate are high would help us to understand this relationship better.

**Fig. 4.**
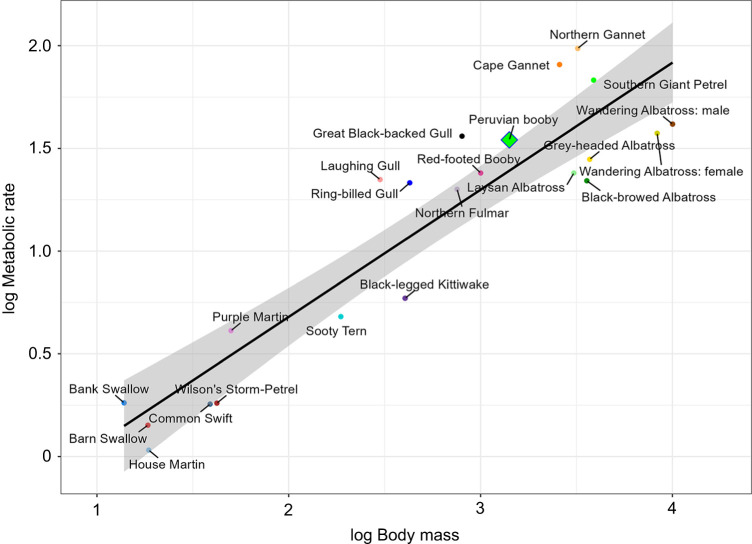
**Multispecies comparison of flight costs of 22 primarily gliding bird species.** Gliding birds were as defined in Guigueno et al. (2019). The metabolic cost of flight was expressed in watts (kJ h^−1^/3.6) and was calculated from activity-specific time budgets, and plotted against body mass (g). Peruvian boobies are indicated as a green diamond. All data were log_10_-transformed. Shaded area represents the confidence intervals.

As observed in other plunge-diving seabirds such as gannets, Peruvian boobies' flight (while commuting) metabolic rate estimated from daily time budgets was higher than those of pure gliders relative to body mass (Fig. 4). However, boobies' flight costs seemed to be much closer to the glider line than those of gannets, implying that they are more efficient flyers, perhaps because they do not flap underwater, allowing them to specialize in flying (Fig. 4). BMR of Peruvian boobies, estimated by the allometric formula from McKechnie and Wolf (2004), was 4.39 W (compared with a value of 0.78 kJ day^−1^ g^−1^ or 12.6 W for our birds ‘at the colony’). Our estimation of commuting flight costs was 7.8×BMR, whereas foraging was 13×BMR, which is consistent with the plunge-diving behaviour of this species, when repeated lifting from the water should be energetically demanding.

Our behavioural classification appeared to reliably partition the daily activity patterns in Peruvian boobies in the PHCS using accelerometry. We restricted our classification to four behaviours thought to be the most important energetically. Potentially, a more refined classification could be achieved by increasing behaviours (searching, resting on water, resting on land, aggressive behaviour at colony, resting at colony, etc.). Nonetheless, our best model included either two sets of two (commuting+foraging and colony+resting) or three grouped behavioural parameters (commuting + foraging + resting away from the colony) of our four initial independent set, which may indicate that modelling DEE may not improve with a greater set of parameters, as noted by Stothart et al. (2016).

Although Peruvian boobies are numerous with a stable population, their high energy requirements – being one of the booby species with the largest clutch sizes, with no signs of siblicide, and one with the highest chick rearing rates, with 1.75 chicks per breeding attempt (Carboneras, 1992) – and more importantly their high foraging costs would explain the slow recovery of their population prior to their collapse in the 1970s in the face of competition with fisheries and lower food availability as a result of climate change. Total time spent away from the colony in boobies is 1.5 times higher during years with harsh conditions (van Oordt, 2024) with an estimated DEE of 0.476 kJ day^−1^ g^−1^ whereas in a normal year it is 0.39 kJ day^−1^ g^−1^. This results in ∼20% energetic increase for Peruvian boobies. Future population models and projections should include accurate energetic requirements for boobies or any species of seabird, as well as the energetic demands associated with climate change and fisheries, which pose a threat to their population stability, recovery and management.

## Appendix

### Additional methods

Results from the LWIA distilled water sample runs were converted into parts per million (ppm) before calculations. We then calculated mean isotope turnover rate for both isotopes, deuterium and oxygen (respectively, *k*_d_ and *k*_o_) with:
(rmA1)

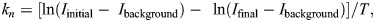
where *k_n_* is the turnover rate for each corresponding isotope, *I*_background_, *I*_initial_ and *I*_final_ represent the isotopes ratios for the corresponding samples, and *T* corresponds to the total sampling time in decimal hours (time between initial and final samples). Because we used the one-sample technique, we deducted the average time of equilibrium from the total sampling time. We then calculated the birds' isotope dilution space of both deuterium and oxygen (*N*_d_ and *N*_o_) using:
(rmA2)

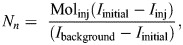
where *N_n_* is the pool for each corresponding isotope, Mol_inj_ represents the total moles of DLW injected into the bird and *I*_inj_ represents the enrichment in ppm of the injectate for each isotope. We assumed a single-pool model for the estimation of CO_2_ production as recommended by Speakman (1997), with boobies being less than 4 kg, using the following reduced formula:
(rmA3)

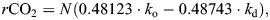
where *r*CO_2_ is the rate of carbon dioxide production (mmol h^−1^) and *N* is the average of *N*_o_ and final dilution space, *N*_f_ (estimated using the ‘percentage mass’ method described by Speakman, 1997). We then converted the resulting value into millilitres of CO_2_ per hour by multiplying by 2240 and used the caloric equivalent of 27.3 kJ h^−1^ assumed for seabirds with diets rich in protein (piscivores) to obtain daily energy expenditure in J. We used daily values instead of total energy values because the latter overestimate relationships (Halsey's time trap; Halsey, 2017). Lastly, as suggested by Stothart et al. (2016), we used mass-specific daily energy expenditure (DEE/mass in grams) values for posterior analyses.

## Supplementary Material

10.1242/jexbio.249555_sup1 Supplementary information
