# Supplementary material for: Plunge-diving into dynamic body acceleration and energy expenditure in the Peruvian booby
Source: J Exp Biol. 2024 Nov 20;227(22):jeb249555. doi: 10.1242/jeb.249555 (PMC11607695; doi:10.1242/jeb.249555)
Supplement: Supplementary information [file jexbio-227-249555-s1.pdf]

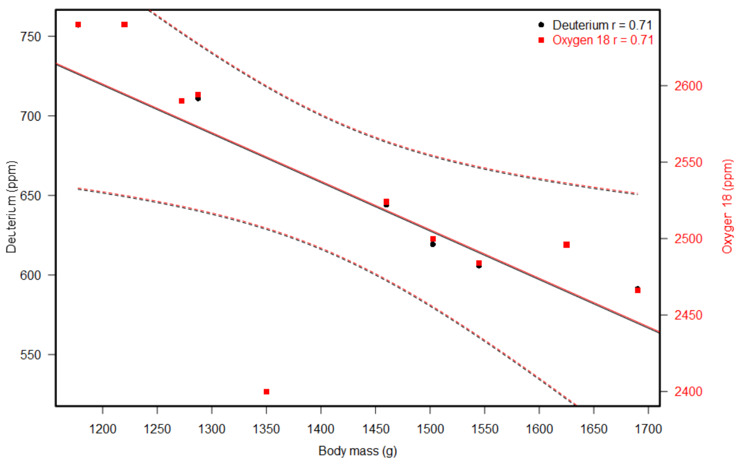

Fig. S1. Relationship of Oxygen-18 and deuterium ^{2}H with body mass after dilution of doubly labelled water for Peruvian boobies at Guañape Norte Island in 2019. Correlation values were obtained from pearson's coefficient (r).

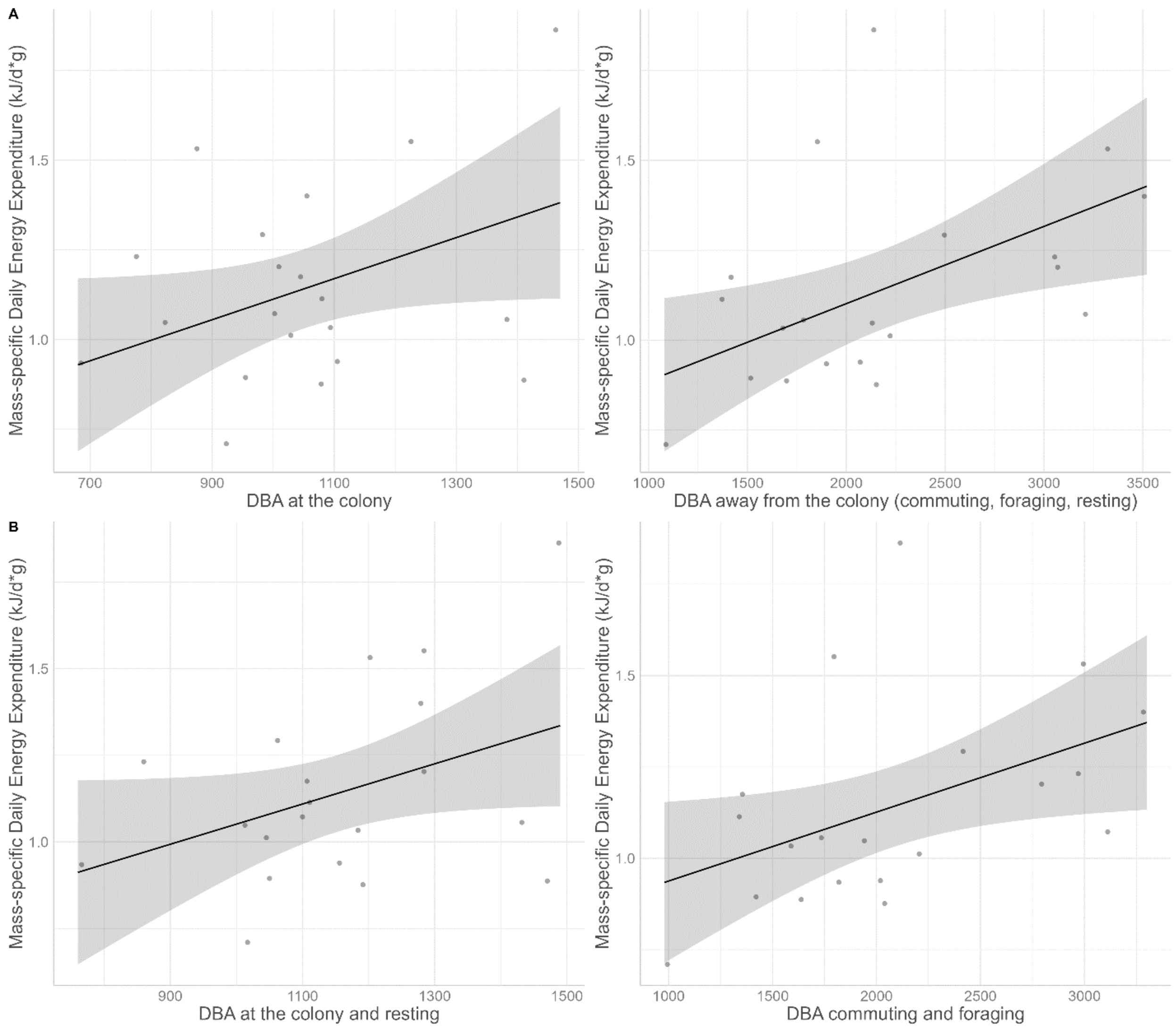

Fig. S2. Model prediction results (marginal effects) for mass-specific DEE from the two best parametrized activity-specific model including: A) DBA at the colony and DBA away from the colony; and B) DBA at the colony and resting, and DBA commuting and foraging.

Journal of Experimental Biology • Supplementary information

Table S1. Full AICc rankings for DEE estimation models for male and female Peruvian boobies.

Model Structure					
Males					
Null	2	-0.44	6.88	0.00	0.69
dailyDBA	3	0.32	10.17	3.28	0.13
TCol + Tcomforrest	3	-0.12	11.04	4.16	0.09
TColRest + TComFor	3	-0.34	11.48	4.59	0.07
DBAColRest + DBAComFor	4	1.02	15.96	9.08	0.01
DBACol + DBAcomforrest	4	0.95	16.10	9.22	0.01
TCol + TRest + TComFor	4	-0.05	18.11	11.22	0.00253
TColRest + TCom + TFor	4	-0.08	18.17	11.28	0.00245
DBAColRest + DBACom + DBAFor	5	1.47	27.06	20.17	0.00003
DBACol + DBARest + DBAComFor	5	1.03	27.95	21.06	0.00002
TCol + TCom + TFor + TRest	5	0.33	29.35	22.46	0.00001
DBACol + DBACom + DBAFor + DBARest	6	1.49	51.01	44.13	<0.00001
Females	df	logLik	AICc	delta	weight
TColRest + TComFor	3	11.59	-13.75	0.00	0.59
TCol + Tcomforrest	3	10.62	-11.81	1.95	0.22
dailyDBA	3	9.10	-8.76	4.99	0.05
TCol + TRest + TComFor	4	11.66	-8.65	5.10	0.05
TColRest + TCom + TFor	4	11.63	-8.60	5.15	0.04
Null	2	6.48	-7.45	6.30	0.03
DBAColRest + DBAComFor	4	10.14	-5.62	8.13	0.01
DBACol + DBAcomforrest	4	9.93	-5.19	8.57	0.01
TCol + TCom + TFor + TRest	5	11.67	-1.34	12.41	0.0012
DBAColRest + DBACom + DBAFor	5	10.34	1.33	15.08	0.0003
DBACol + DBARest + DBAComFor	5	10.18	1.64	15.39	0.0003
DBACol + DBACom + DBAFor + DBARest	6	10.35	12.30	26.06	0.000001

Table S2. Model estimates for best two parametrized models estimating mass-specific DEE from AICc rankings for Peruvian Boobies (sexes grouped).

Model for DBA at the colony and resting and DBA commuting and foraging

	Estimate	Std. Error	t	p
(Intercept)	4.091e-02	3.745e-01	0.109	0.9143
$DBA_{col+rest}$	2.376e-06	1.134e-06	2.095	0.0515
$DBA_{com+for}$	7.583e-07	3.264e-07	2.323	0.032

Model for DBA at the colony and DBA away from the colony

(Intercept)	4.062e-02	3.798e-01	0.107	0.9161
DBA_{col}	2.334e-06	1.129e-06	2.067	0.0543
$DBA_{com+for+rest}$	8.858e-07	3.109e-07	2.850	0.0111

Table S3. Activity-specific time budget and DBA values for all Peruvian boobies in Guañape island in 2019.

Activity	Mean Time	Hours per day	SD	Min	Max
Colony	0.80	19.23	0.08	0.53	0.89
Commuting	0.10	2.32	0.04	0.05	0.19
Foraging	0.06	1.54	0.01	0.04	0.09
Resting	0.04	0.91	0.05	0.01	0.25

	Mean DBA	SD	Min	Max
Colony	259808.4	48718.6	170661.9	350979.7
Commuting	268628.2	115096.9	124748.1	481935.3
Foraging	252140.2	64817.7	122618.7	369233.1
Resting	37008.2	22789.9	11505.4	98245.5

Journal of Experimental Biology • Supplementary information
